# Combining a New Exome Capture Panel With an Effective varBScore Algorithm Accelerates BSA-Based Gene Cloning in Wheat

**DOI:** 10.3389/fpls.2020.01249

**Published:** 2020-08-13

**Authors:** Chunhao Dong, Lichao Zhang, Zhongxu Chen, Chuan Xia, Yongqiang Gu, Jirui Wang, Danping Li, Zhencheng Xie, Qiang Zhang, Xueying Zhang, Lixuan Gui, Xu Liu, Xiuying Kong

**Affiliations:** ^1^Key Laboratory for Crop Gene Resources and Germplasm Enhancement, MOA, National Key Facility for Crop Gene Resources and Genetic Improvement, Institute of Crop Sciences, Chinese Academy of Agricultural Sciences, Beijing, China; ^2^Department of Life Science, Chengdu Tcuni Technology, Chengdu, China; ^3^State Key Laboratory of Crop Gene Exploration and Utilization in Southwest China, Sichuan Agricultural University, Chengdu, China; ^4^Triticeae Research Institute, Sichuan Agricultural University, Chengdu, China; ^5^Western Regional Research, United States Department of Agriculture-Agricultural Research Service, Albany, CA, United States

**Keywords:** wheat exome capture, varBScore, gene cloning, bulked segregant analysis, CRISPR/Cas9, yellow-green leaf mutant

## Abstract

The discovery of functional genes underlying agronomic traits is of great importance for wheat improvement. Here we designed a new wheat exome capture probe panel based on IWGSC RefSeq v1.0 genome sequence information and developed an effective algorithm, varBScore, that can sufficiently reduce the background noise in gene mapping and identification. An effective method, termed bulked segregant exome capture sequencing (BSE-Seq) for identifying causal mutations or candidate genes was established by combining the use of a newly designed wheat exome capture panel, sequencing of bulked segregant pools from segregating populations, and the robust algorithm varBScore. We evaluated the effectiveness of varBScore on SNP calling using the published dataset for mapping and cloning the yellow rust resistance gene *Yr7* in wheat. Furthermore, using BSE-Seq, we rapidly identified a wheat yellow leaf mutant gene, *ygl1*, in an ethyl methanesulfonate (EMS) mutant population and found that a single mutation of G to A at 921 position in the wild type *YGL1* gene encoding magnesium-chelatase subunit chlI caused the leaf yellowing phenotype. We further showed that mutation of *YGL1* through CRISPR/Cas9 gene editing led to a yellow phenotype on the leaves of transgenic wheat, indicating that *ygl1* is the correct causal gene responsible for the mutant phenotype. In summary, our approach is highly efficient for discovering causal mutations and gene cloning in wheat.

## Introduction

Wheat (*Triticum aestivum* L., 2n = 6x = 42, AABBDD) is an important staple crop, providing 20% of all calories consumed by the world population. Identification of genes underlying desirable agronomic traits is of great significance for genetic improvement of wheat. However, wheat functional genome research has lagged behind model plant species due to the lack of complete reference genome sequence in previous years and its complex hexaploid genome. The release of genome sequences of several wheat varieties and progenitors provides an opportunity to explore the rich genetic resources of wheat for crop improvement (Avni et al., 2017; Luo et al., 2017; Zhao et al., 2017; Appels et al., 2018; Ling et al., 2018; Maccaferri et al., 2019).

Dissecting the genetic and molecular mechanisms regulating crop yield and growth is of great importance for crop improvement. Screening of induced mutant populations is an effective approach for discovering new genes underlying phenotypic variations in plants (Schneeberger, 2014; Krasileva et al., 2017). As a traditional forward genetic method, map-based cloning has been widely used to clone genes controlling traits of interest. Using map-based cloning approaches, several very important genes, such as vernalization (Yan et al., 2003; Yan et al., 2004), male sterility (Ni et al., 2017; Xia et al., 2017), and resistance genes (Fu et al., 2009), have been cloned. However, map-based gene cloning usually requires multiple steps, including generating mapping populations, fine mapping to narrow the target gene region to identify genetic markers co-segregating with the target phenotype, candidate gene screening and gene identification by sequencing. This process is often time-consuming and costly, especially in wheat.

With the great advances of next generation sequencing technologies, it is now feasible to dissect all genetic variants within a mutant line and then identify the causal variants associated with the target phenotype through combining bulked segregant analysis (BSA) with the whole genome sequencing strategy (Michelmore et al., 1991; Schneeberger, 2014). Mapping-by-sequencing has been widely implemented in model plants Arabidopsis and rice to screen the causal mutations underlying corresponding phenotypes from ethyl methanesulfonate (EMS) mutant populations (Austin et al., 2011; Lim et al., 2014; Schneeberger, 2014). To map the causal mutation, high-throughput sequencing is applied on bulked DNA samples pooled from individuals sharing the mutant and wild phenotype from an F_2_ population derived from the cross between the mutant line and a genetically divergent accession or backcross line (Schneeberger et al., 2009; Austin et al., 2011; Schneeberger, 2014). The successful application of methods such as MutMap, MutMap^+^, MutMap-Gap, SHOREmap and MMAPPR proved that combining BSA with high-throughput sequencing (BSA-Seq) is a feasible alternative to map-based cloning for new gene discovery (Schneeberger et al., 2009; Abe et al., 2012; Fekih et al., 2013; Hill et al., 2013; Takagi et al., 2013; Takagi et al., 2015; Yuan et al., 2017).

Although improved high-throughput sequencing technologies have significantly reduced cost and increased data quality, it is still too expensive to generate whole-genome sequence data for wheat due to its large genome size and highly repetitive content (Choulet et al., 2010). To overcome this limitation, exome capture technologies aiming to cover the majority of the gene coding regions have been developed (Henry et al., 2014; Krasileva et al., 2017; He et al., 2019). Several wheat exome capture probe sets have been developed based on the previously released draft Chinese Spring genome sequence data (Krasileva et al., 2017). Recently, 890 diverse accessions of hexaploid and tetraploid wheat were re-sequenced through exome capture technology to reveal that wild-relative introgression through historic gene flow has made a significant contribution to the adaptive diversity of modern wheat (He et al., 2019). Exome capture has also been used for mapping and gene identification in wheat. A 110-Mb exome capture assay was used to identify candidate natural variants at the *Yr6* locus responsible for yellow rust resistance through mapping-by-sequencing (Gardiner et al., 2016). In addition, an 84-Mb exome capture assay was employed to uncover the coding region mutations in EMS-induced TILLING populations containing 2,735 mutant lines of tetraploid and hexaploid wheat (Krasileva et al., 2017). Using bulked segregant analysis and the exome sequence strategy, Harrington et al. identified a locus controlling a dominant, environmentally dependent chlorosis phenotype within the Durum wheat (*Triticum turgidum*) cv. Kronos (Harrington et al., 2019), Mo et al. (2018) identified a clear peak region on chromosome arm 4BS associated with increased plant height, Martinez et al. (2020) fine-mapped the ABA-hypersensitive mutant ERA8 in a wheat backcross population. Using RenSeq and MutRenSeq strategy, series resistance genes were cloned through developing the exome capture probe panel that specifically captures disease resistance (R) genes (Jupe et al., 2013; Steuernagel et al., 2016). Moreover, AgRenSeq was further developed in exploiting pan-genome variation and rapid R gene cloning by combining association genetics with R gene enrichment sequencing (Arora et al., 2019).

Integrating recently developed genome sequence and annotation resources, Gardiner et al. designed two gold standard capture probe sets for hexaploid bread wheat, with the final gene/putative promoter design space of 785,914,746 bp of which 508,889,665 bp was gene and 277,025,081 bp was putative promoter sequences (Gardiner et al., 2019). The application of exome capture assays not only provides us with a fast and convenient method to study domestication and evolution of wheat, but can also generate coding sequence information of a large number of genetic materials that can facilitate reverse genetic screening of a mutant line with known mutation sites. Currently, most wheat exome capture probe sets were designed prior to the recent release of high-quality reference genomes, with a large amount of sequences likely uncovered in the exome capture panels. Therefore, a new exome capture panel with a higher sequence coverage using the latest genome information would be more useful in a wide range of applications.

Leaf color has been extensively studied in rice and other plants because of its large effect on yield by affecting photosynthesis and is an efficient phenotypic marker in hybrid crops (Zhang et al., 2018). However, genetic and molecular characterization of leaf color genes are very limited in wheat, largely due to its genome complexity and high proportion of repetitive sequences. Most studies have focused on phenotypic characteristics, physiological and biochemical characteristics, and genetic mapping (Wu et al., 2018). In this study, we identified and characterized a yellow-green leaf mutant (*ygl1*) from a wheat EMS mutant population. To identify the causal mutation responsible for the phenotype, we designed a new wheat exome capture platform and developed an efficient statistical analysis model, named varBScore, to perform the BSA-Seq analysis and rapidly clone the *YGL1* gene responsible for the yellow-green leaf phenotype in wheat.

## Materials and Methods

### Plant Materials

The yellow-green leaf mutant *ygl1* originated from mutagenesis of 6,000 seeds of the elite wheat variety YZ4110, which was treated with 1.2% EMS; the germination rates of the EMS-mutagenized seeds were 65% (Zhao et al., 2009), and the yellow-green leaf phenotype was inherited stably after four generations of self-pollination. For the genetic analysis and/or gene mapping, *ygl1* was backcrossed with YZ4110 to produce a BC_3_F_2_ population consisting of 1,204 individuals. *ygl1* was also crossed with a genetically divergent accession Bainong3217, generating an F_2_ population that consisted of 431 individuals. The phenotype of all F_2_ plants was further validated by using F_3_ families. Wheat plants were cultivated in the experimental field of the Institute of Crop Sciences, Chinese Academy of Agricultural Sciences, Beijing, China. In addition, all the transgenic plants were grown in a growth chamber maintained at 24°C, 16/8 h light/dark with 300 μ mol m^−2^ s^−1^ light intensity at 45% humidity.

### Chlorophyll and Carotenoid Content Analysis, and Transmission Electron Microscopy Analysis

The content of chlorophyll and carotenoid was determined with a DU 800 UV/Vis Spectrophotometer according to the method described by Sheng et al. (2017), each sample included ten biological replicates. Chloroplast structures were examined in the four-week-old leaf samples of the wild-type YZ4110 and *ygl1* mutant plants using transmission electron microscopy. Leaves were cut to sections approximately 5 mm in length and fixed in a 2% glutaraldehyde solution in phosphate buffer. Subsequently, the sections were fixed in a 1% OsO_4_ solution, and the samples were stained with uranyl acetate, followed by dehydration through serial ethanol dilution. The thin sections were embedded in Spurr’s medium. Finally, the samples were sliced to 50 nm in thickness and stained again, and then viewed with a JEOL 100 CX electron microscope.

### Exome Capture Design

The capture probe sequences were mainly based on the RefSeq Annotation v1.0, covering 107,891 high-confidence (HC) protein-coding genes and 161,537 low-confidence (LC) genes, 5′UTR and 3′UTR sequences were included. To capture exons as much as possible in the wheat genome, over 2,600 RNA-seq data downloaded from Sequence Read Archive (SRA) database of the National Center for Biotechnology Information (NCBI) (https://www.ncbi.nlm.nih.gov/sra/) were aligned to the IWGSC CS RefSeq v1.0 genome with STAR(v2.6.1b) (Dobin et al., 2013). The novel transcriptome was assembled with Stringtie (v.1.3.5) (Pertea et al., 2015) based on IWGSC annotation v1.1 with both HC and LC genes. The novel transcriptome (TMP >2) was used to predict ORFs using TransDecoder (v5.5.0) (Haas et al., 2013) with default parameters. All CDS and untranslated regions (UTR) of novel and HC/LC genes were extracted as redundancy capture targets sets. Basically, a histogram of the frequency of 15mers within the reference genome was constructed, and candidate probes were assigned a score based on probe sequence matches to all 15mers. Probes with scores higher than 100 were rejected. This set contained probes with up to 20 close matches to the genome, as determined by the SSAHA algorithm (Ning et al., 2001), for the purposes of providing better coverage. A probe was aligned to the genome if there were a total of five or fewer variations including single-base insertions, deletions or substitutions between the probe and the genome sequences.

### Exome Capture Sequencing

The genomic DNA of 30 extreme phenotype individuals from different lines of the Bainong3217/*ygl1* F_3_ population was extracted by means of CTAB method (Chatterjee et al., 2002), and then the genomic DNA was bulked in an equal ratio to generate the wild-type bulked DNA pool and mutant-type bulked DNA pool separately. A total of 4.5 mg genomic DNA (35 ng/ml) was fragmented into 200 bp using the Bioruptor UCD-200 sonicator (Diagenode, Denville, NJ). Pre-libraries were constructed using the Kapa Hyper DNA library prep kit for Illumina HiSeq. Fragmented DNA was end-repaired with an end-repair enzyme, and a deoxyadenosine was added to the 3′ ends of the fragments. Kapa barcoded DNA and Kapa indexed adapters were ligated to the sample libraries. The adapter-ligated libraries were selected for an average insert size of 200 bp using next-generation sequencing cleanup and size select (NucleoMag, Macherey-Nagel, Duren, Germany) according to the manufacturer’s instructions. The pre-capture amplification was performed using the NimbleGen protocol with eight PCR cycles. Hybridization of sample libraries was performed at 47°C for 60–72 h using the SeqCap EZ library (Roche/NimbleGen, Madison, WI) and custom-designed exome capture probes. The hybrids were enriched using the capture beads from the SeqCap EZ pure capture bead kit, washed, and amplified by ligation-mediated PCR. The quality of the captured libraries was assessed using the Bioanalyzer 4200 (Agilent Technologies, Santa Clara, CA). The libraries were quantified by qRT-PCR and then sequenced using the Illumina HiSeq Nova platform to generate 150-bp paired-end reads.

### The BSE-Seq Pipeline for Rapid Gene Cloning

**Figure 1:**
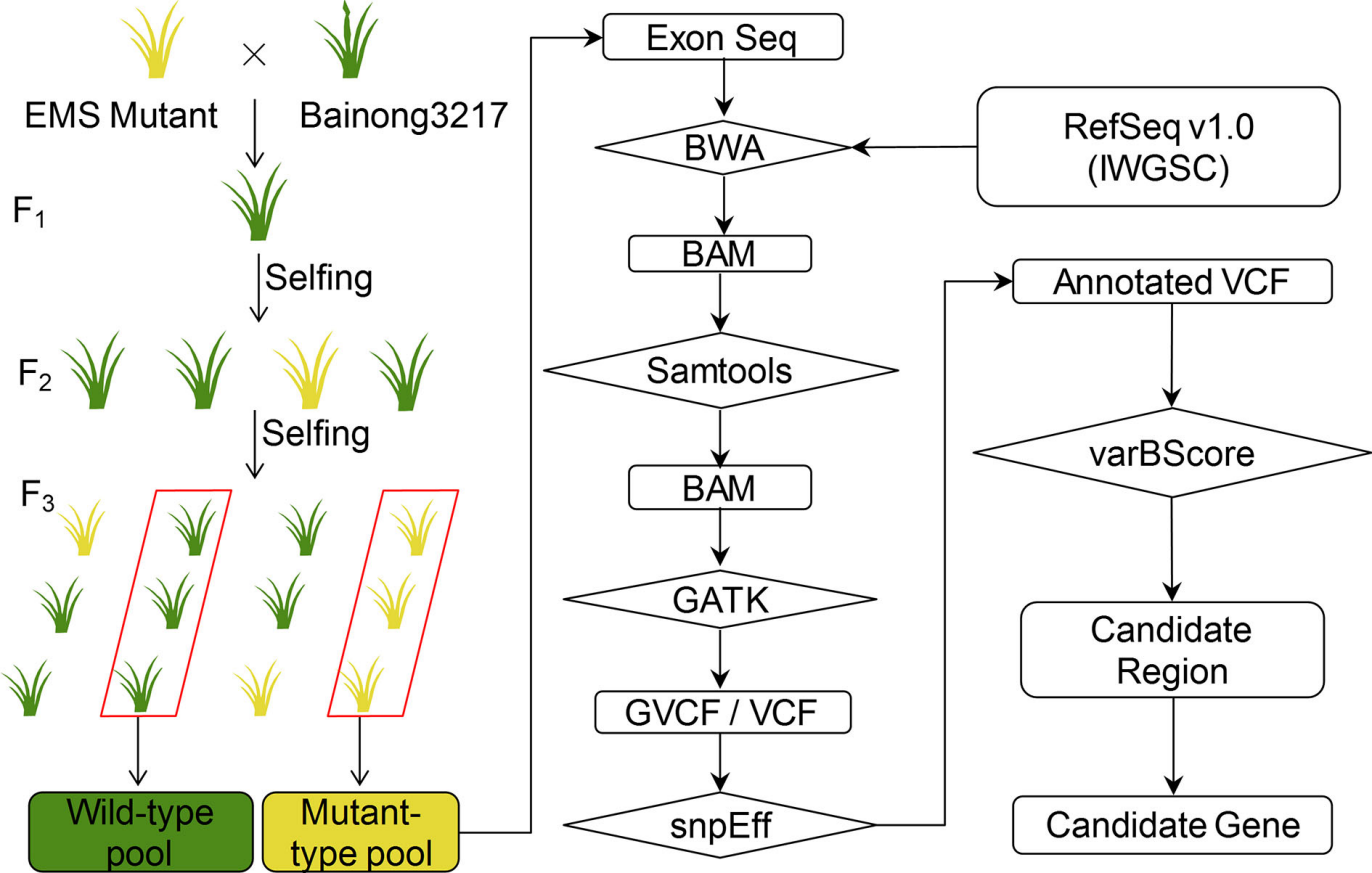
Flow chart of the BSE-Seq analysis method. The seeds of a wheat cultivar were mutagenized by ethyl methanesulfonate (EMS). Then, the mutant was crossed to the genetically divergent accession Bainong3217. The resulting F_1_ plants were self-pollinated to obtain F_2_ segregating progeny. DNAs of F_3_ displaying the wild-type and mutant phenotypes were bulked and subjected to exome capture sequencing followed by alignment to the reference sequence. Then, the SNPs were called and analyzed to identify the causal SNP for the mutant phenotype.

With the newly designed exome capture probe panel and varBScore, we developed a BSA-based exome capture sequencing (BSE-Seq) pipeline for rapid gene cloning in wheat. This approach consists of the following steps (**Figure 1**): (1) Develop a population derived from the cross between the mutant line and a genetically divergent accession or a backcross to its parental wild-type plant line. (2) DNAs from the two selected groups showing extreme phenotypes are extracted and bulked in equal amounts, forming two sample pools. (3) The two DNA pools are subjected to exome capture sequencing with deep coverage (~70×); raw sequencing reads are processed using Fastp (v0.12.4) to remove low quality reads and adapter sequences. (4) BWA (v0.7.16) mem is applied to align the high-quality reads to the IWGSC RefSeq v1.0 genome with default parameters. (5) Samtools (v1.9) is used to BAM sort, remove PCR duplicated reads and generate sample alignment statistics. (6) The raw cohort vcf is analyzed with GATK (v4.0.10.1) module. The minimum-mapping-quality parameter is set as 30 for high-quality alignment reads that are used to call variants. (7) bcftools (v1.9) is performed as variants quality filtering with “QUAL>30” and “DP≥5”. To annotate variants, customized databases containing IWGSC v1.1 HC/LC genes and annotated genes are created by snpEff (v4.3T). (8) The new statistical model varBScore is then introduced to accurately determine the position of the target candidate gene. (9) The 2,600 RNA-seq data were used as a “background” to identify the causal mutation based on the assumption that the genetic materials carrying the same mutation may have the same phenotype. Using the yellow-green leaf color mutant *ygl1* used in this study as an example, we searched the candidate mutants in the background which is composed of materials with green leaf phenotype. As result, the mutations that appeared in the background would be filtered out, which is helpful in identifying the causative mutation.

### Data Processing

Raw sequence reads were processed using Fastp (v0.12.4) (Chen et al., 2018) to remove low quality reads and adapters. BWA (v0.7.16) (Li and Durbin, 2009) mem was applied to align the high-quality reads to IWGSC RefSeq v1.0 genome with default parameters. Samtools (v1.9) (Li et al., 2009) was used to bam sort, remove read PCR duplications and for sample alignment statistics. After that, raw cohort vcf was worked out with GATK (v4.0.10.1) (McCormick et al., 2015) module BaseRecalibrator, ApplyBQSR, HaplotypeCaller, CombineGVCFs and GenotypeGVCFs were used. The minimum-mapping-quality parameter was set as 30 for only high-quality alignment reads used to call variants. bcftools (v1.9) (Narasimhan et al., 2016) was performed for variants quality filtering with “QUAL > 30” and “DP >= 5”. To annotate variants, customized databases containing IWGSC v1.1 HC/LC genes were generated by snpEff (v4.3T) (Cingolani et al., 2012).

### Phylogenetic Analysis

All the protein sequences were obtained from Ensembl Plants (http://plants.ensembl.org/Triticum_aestivum/Tools/Blast) using the YGL1 protein sequence as a query, and the homoeologs that shared more than 60% identity with YGL1 were selected for analysis. After alignment of amino acid sequences by ClustalW with default parameters, the phylogenetic tree was constructed using MEGA 7.0 (Kumar et al., 2016) based on the neighbor-joining method, and the bootstrap values were estimated with 1,000 replicates.

### Transgene Construction

The target sites of guide RNA were designed by a web-based toolkit E-CRISP (Heigwer et al., 2014). A binary vector *TaU3p-413* that can carry two guide RNAs and the intermediate vector *pCBC-MT1T2* were used to create the CRISPR construct (Xing et al., 2014) by the Golden gate method (Engler et al., 2008). Briefly, the target site (5′-CAACAGGGGGATACTGTATG-3′) was incorporated into both PCR forward and reverse primers (**Supplementary Table S3**). Then the PCR fragment with the target site that was amplified from *pCBC-MT1T2* was assembled into the binary vector *TaU3p-413* using *Bsa*I and T4 Ligase (New England Biolabs). Then the plasmid was transformed into the *Agrobacterium tumefaciens* strain EHA105 and introduced into immature embryos of the wheat cultivar ‘Fielder’ *via* an *Agrobacterium tumefaciens-*mediated transformation method using licensed protocols of ‘PureWheat’ (Ishida et al., 2015).

## Results

### Development of a New Wheat Exome Capture Panel

The exome capture panel was developed based on the IWGSC RefSeq v1.0 sequence information (Appels et al., 2018) and the Roche NimbleGen probe synthesis technology (OID46587, IRNSOW1123). A total of 299,387,477 bp regions were selected in the probe design, mainly including two aspects: (1) the 107,891 High Confidence (HC) and 161,537 Low Confidence (LC) genes containing the sequences from the 5′UTR (untranslated region) to the 3′UTR, which come from RefSeq Annotation v1.1, and (2) novel transcripts annotated from 2,600 RNA-seq data downloaded from the Sequence Read Archive (SRA) database of the National Center for Biotechnology Information (NCBI) (https://www.ncbi.nlm.nih.gov/sra/); these transcripts were highly expressed but beyond the scope of RefSeq Annotation v1.1. Approximately 54,241,943 bp regions were absent due to the unused sequence including gaps and sequence repeats, and a final probe length of 245,145,534 bp was accumulated. In addition, a single 120 bp probe can enrich up to 500 bp routinely with adequate sequencing coverage, with the flanking sequence besides the exons could be captured simultaneously (Gardiner et al., 2015), the coverage length is approximately 277,596,390 bp estimated by a 200 bp fragment library (**Table 1**). We aligned the 245,145,534 bp probe sequences to the reference genome sequences from IWGSC annotation v1.1 and identified 107,400 high confidence genes and 132,688 HC transcripts.

**Table 1:** Summary of the new designed 260 Mb exome capture panel.

Genome	IWGSC_ReferSeq_v1.0	
Number of regions	680,432	
Length of regions (bp)	299,387,477	
Statistics	Probe Coverage	Estimated Coverage
Target Bases Covered	245,145,534	277,596,390
% Target Bases Covered	81.88	92.72
Targets with no coverage	37,897	36,717
Target Bases Not Covered	54,241,943	21,791,087
Due to sequence gaps	144,188	64,900
Due to repeats	21,620,096	142,93,102
% Target Bases Not Covered	18.12	7.28
Due to sequence gaps	0.05	0.02
Due to repeats	7.22	4.77
Total capture targets	1,806,109	
Total capture space (bp)	268,933,489	

### Rationale Behind varBScore

One of the challenges of sequencing-based methods to identify causal mutation sites is the high background noise, attributable to the large number of resulting potential SNPs and sequencing/mapping errors, particularly in large and complex genomes such as wheat. We developed a new sequence data analysis model, varBScore, which calculates the variance between the observed allele frequency and the expected allele frequency in a sliding window, which could reduce the noise in the process of mapping the causative mutation to a large extent.

Theoretically, according to the Mendelian inheritance, for a population generated from the selfing of a heterozygous parent which carries plenty of SNPs, every SNP locus has a distribution of AA : Aa:aa = 1:2:1. For instance, a stable EMS-induced mutant which is controlled by a recessive gene crossed with its wild-type and generating a heterozygote, then producing an F_2_ population by selfing, can be separated into two pools by phenotype: the wild-type pool, which contains homozygotes (AA) and heterozygotes (Aa), and the mutant-type pool, composed of homozygous mutants (aa). The expected allele frequency of the causative mutation in wild-type pool (AA : Aa = 1:2) would be 1/3; similarly, its expected allele frequency in mutant-type pool (aa) would be 1. The expected allele frequency of other SNPs would be related to their linkage to the causative mutation, and those SNPs that have a close linkage with the causative mutation may share the similar expected allele frequency with the causative mutation. The varBScore algorithm screens the segments that meet the expected allele expectations by calculating the Standard Deviation (SD) between the expected allele frequency and the observed allele frequency of every SNP.

The varBScore can be calculated as follows:

$$\begin{array}{l} \mathrm{var}\;BScore_{k}^{j}=-\log10\left(\sqrt{\frac{+{\left(M_{k}^{j}-\bar{m}\right)}^{2}}{n-1}}+c\right)\\ {}*-\log10\left(\sqrt{\frac{+{\left(W_{k}^{j}-\bar{w}\right)}^{2}}{n-1}}+c\right) \end{array}$$

Frequency of alternate alleles (AF) different > *df* between wild type and mutant type, $df=|M_{k}^{j}-W_{k}^{j}|$.

$M_{k}^{j}$: *j* locus AF of mutant bulk; $\bar{m}$: expectation AF of mutant bulk; $W_{k}^{j}$: *j* locus AF of wild bulk; $\bar{w}$: expectation AF of wild bulk; *n*: number of SNP in windows *k*; *c* = 0.01, *c* = 0.001, *c* = 0.0001…;

### Validation of varBScore on a Known Mutant in Wheat

*Yr7* is a major Yellow stripe rust (YR) resistance gene that is identified in wheat. Gardiner et al. (2020) firstly used a traditional QTL mapping method and mapped *Yr7* to a low-recombination region near the centromere of Chromosome 2B by a double haploid (DH) population, then the association mapping panel and mapping-by-sequencing strategy were then conducted to fine map the *Yr7* gene. Finally, a 60 Mb region that contains the *Yr7* locus was identified on chromosome 2B (633,430,001–663,920,001 bp, 681,790,001–693,030,001 bp and 702,660,001–721,190,001 bp). The candidate region contains 589 high confidence genes; 10 disease resistance genes that are mainly clustered together in the region 683,043,955–686,815,417 bp (~4 Mbp) are considered as candidate genes for *Yr7* resistance.

**Figure 2:**
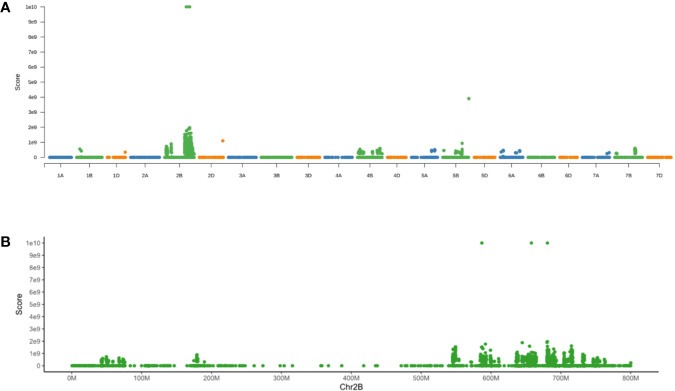
Results for mapping of *Yr7* by varBScore. **(A)** Manhattan plot of the varBScore along each chromosome. **(B)** Manhattan plot of the varBScore along chromosome 2B.

To test the varBScore algorithm, we download the BSA data set of *Yr7* (https://www.ebi.ac.uk/ena, study PRJEB36010) and then analyzed the data by using the varBScore algorithm, which showed a very distinct peak on chromosome 2B along each chromosomal pseudomolecule (**Figure 2A**). Further analysis showed that the highest scoring points were located on the regions (586,775,469–88,621,811 bp, 655,257,794–658,193,278 bp, and 680,759,276–682,847,904 bp) of chromosome2B (**Figure 2B**), and the third peak region was close to the candidate region. In our result, the candidate gene could be mapped to such a region without any other approaches, indicating that varBScore could be effective on gene mapping.

### Rapid Cloning of a Wheat Yellow-Green Leaf Gene *YGL1*

**Figure 3:**
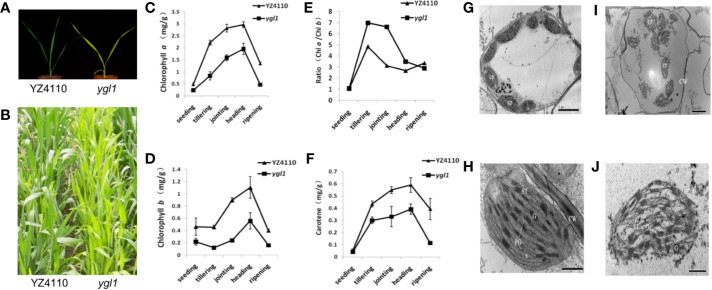
Characterization of wheat yellow-green leaf mutant *ygl1*. **(A, B)** Phenotype of YZ4110 and the *ygl1* mutant at seedling and mature stage, respectively; **(C, F)** Chlorophyll *a* **(C)**, Chlorophyll *b* **(D)**, the ratio of Chlorophyll *a* and Chlorophyll *b* **(E)** and carotene contents **(F)** in leaves of the YZ4110 and the *ygl1* mutant; Data indicate average ± standard deviation (SD) (n = 10). **(G, J)** Transmission electron microscopy analysis of chloroplasts in the YZ4110 **(G**, **H)** and the *ygl1* mutant **(I, J)**. CP, chloroplast; PG, plastoglobule; G, grana; S, stroma; CW, cell wall; ST, stroma thylakoid. Scale bar = 5 μm in G and H, scale bar = 1 μm in **(I, J)**.

To further verify the effectiveness of BSE-Seq, a chlorotic leaf phenotype mutant *ygl1*, obtained by ethyl methanesulfonate (EMS) mutation of wheat variety Yanzhan4110 (YZ4110), was used to conduct the analysis. Compared with the wild type, *ygl1* exhibited obvious yellow leaf color from seedling to mature stage (**Figures 3A, B****)**. The contents of chlorophyll *a* (Chl *a*), chlorophyll *b* (Chl *b*), and carotene were significantly reduced (**Figures 3C–F**). Transmission electron microscopy (TEM) analysis revealed that the shape and size of the chloroplast of the wild type and the mutant were not significantly different. However, in the mutant, the outer membrane structures of many chloroplasts are broken, the inner chloroplast matrix and thylakoid are exposed, and the number of normal chloroplasts is less than that of wild-type plants (**Figures 3G–J**). Genetic analysis of 1,204 BC_3_F_2_ population plants derived from a backcross between the mutant line *ygl1* and wild-type YZ4110 showed that the yellow leaf phenotype of *ygl1* was controlled by a recessive nuclear gene (**Supplementary Table S1**).

**Figure 4:**
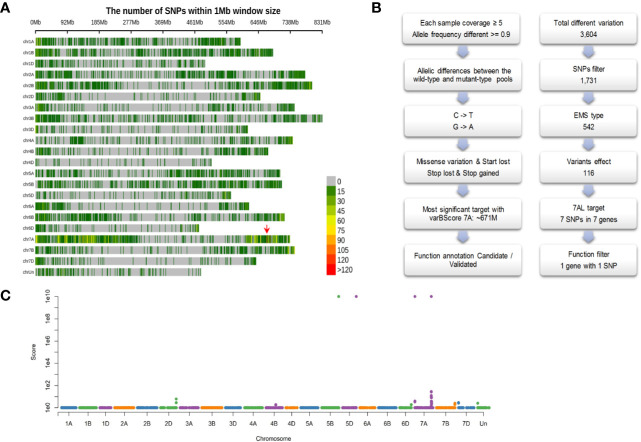
Identification of the candidate causal mutation. **(A)** The distribution of SNPs and significant candidate selected region (indicated with red arrow) across the 21 wheat chromosomes; **(B)** The principle of filtering variation based on EMS mutagenesis characteristics; **(C)** Manhattan plot of varBScore along each chromosomal pseudomolecule.

We first developed a F_2:3_ segregating population (431 individuals) derived from the cross between *ygl1* and a genetically divergent accession Bainong3217. The yellow and green pools bulked from the homozygous genotype of 30 yellow and 30 green F_3_ individual plants were constructed, respectively. Exome capture and high-throughput sequencing were conducted and generated approximately 20 Gb of sequence data for each pool. Compared with the Chinese Spring reference genome, a total of 777,780 and 792,839 sequence variations were detected in yellow and green pools, respectively. By projecting these variations to chromosomes, we found that the most significant varBScore was at the 671 Mb position on chromosome 7A (**Figure 4A**).

**Figure 5:**
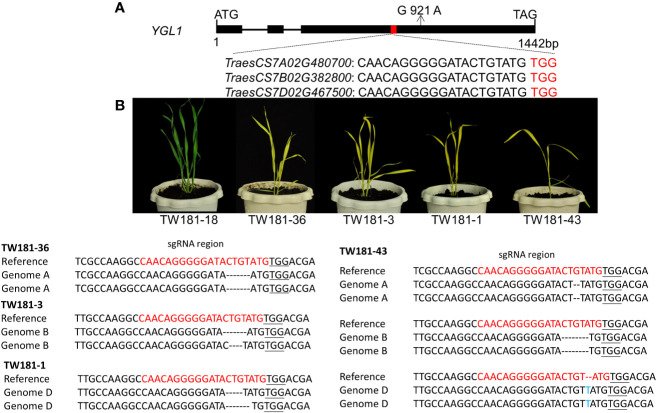
Cloning and functional validation of *YGL1*. **(A)** Gene structure of the *YGL1*. The black boxes in the gene structure represent exons, the lines between them represent introns, the red box represents conserved region of *YGL1* homoeologs, sequences below represent the sgRNA target loci, and the red-colored letters are PAM motifs. **(B)** Phenotypes and genotypes of the transgene but edited negative (TW181-18) and homozygous/bi-allelic edited T_0_ plants. Nucleotide sequences in red show the sgRNA region; sequence underlined indicates the PAM sequence, black dashes in the sequence represent the deletions, and letters in blue represent insertions or substitutions.

Given the characteristics of EMS mutagenesis, certain variations should be filtered out based on the following criteria (**Figure 4B**): (1) Allelic differences between the yellow and green pools. A total of 3,604 SNPs were discovered, and the SNPs that had allelic differences (the green pool had an identical genotype with the reference genome, and the yellow pool had a mutant genotype, or reverse) between pools were 1,731. (2) Because EMS-type mutations (base changes from C to T or G to A) constitute over 99% across the population (Krasileva et al., 2017), approximately 542 EMS-type mutations were retained. (3) Omitting mutations that could lead to missense, loss of start codon, loss of stop codon and gain of coding amino acids, and 116 SNPs were left. (4) Further screening was conducted by filtering out the SNPs that could be detected in the “background”, which is comprised of the materials with green leaf; this step could filter out the SNPs generated from the sequencing errors, mapping errors, homoeologous genes and the same variations existing in the genetic materials. Through the above filtering steps, we finally obtained the seven most significant SNPs on chromosome 7A at the 671 Mb position (**Figure 4C**, **Table 2**). Functional annotation of the genes carrying these seven SNPs (**Table 2**) revealed that *TraesCS7A02G480700* (**Figure 5A**) encoding the chloroplast magnesium-chelatase subunit chlI, a key enzyme in chlorophyll synthesis and chloroplast development, may be involved in the regulation of leaf color. Sequence analysis indicated that *TraesCS7A02G480700* encodes an ortholog of *OsChlI*, whose single-recessive mutants exhibiting a yellowish-green leaf color (Zhang et al., 2006) (**Supplementary Figure S1**), which has a similar phenotype with the *ylg1* mutant.

**Table 2:** Candidate genes and nucleotide variations of targets with the most significant varBScore (Chr7A: ~671 M).

Chr	Pos	Reference	mutant base	Feature	Gene	Wildtype Pool	Mutant Pool	Functional Annotation
chr7A	630,870,068	G	A	Missense variant	TraesCS7A02G436400	5|0	0|5	Ethylene-responsive transcription factor
chr7A	655,451,742	C	T	Missense variant	TraesCS7A02G460100	31|0	2|32	Protease inhibitor/seed storage/lipid transfer protein family protein
chr7A	66,2134,333	C	T	Missense variant	TraesCS7A02G465600	11|0	0|6	Ubiquitin family
chr7A	662,143,366	C	T	Missense variant	TraesCS7A02G465700	38|0	1|19	Transcription factor FapR
chr7A	672,873,436	C	T	Missense variant	TraesCS7A02G480700	34|0	0|53	Mg-protoporphyrin IX chelatase
chr7A	680,628,523	C	T	Missense variant	TraesCS7A02G492000	18|0	1|10	ATP-dependent zinc metalloprotease
chr7A	682,175,198	C	T	Missense variant	TraesCS7A02G493600	4|0	0|4	Heat shock family protein

We further examined the genotypes of these seven SNPs in both parents and progenies of the backcross population. The results showed that the genotype of *TraesCS7A02G480700* in wild-type YZ4110 and 43 homozygous F_3_ individual plants with green leaves was G at the position 921, while it was A in the mutant *ygl1* and 34 homozygous F_3_ individual plants with yellow leaf phenotype. The rest of the SNPs have some recombination events among the progenies, which means they are not casual mutations.

### Verification of the Candidate Gene *YGL1* Through Genome Editing With CRISPR/Cas9

A genome-editing experiment using the CRISPR/Cas9 system was further implemented; a single guide RNA was designed to target the site within a conserved region of three *YGL1* homoeologous genes (*TraesCS7A02G480700*, *TraesCS7B02G382800*, *TraesCS7D02G467500*) on chromosomes 7A, 7B, and 7D genomes (**Figure 5A**); the primers used for vector construction and editing identification are listed in **Supplementary Table S3**. In the transgenic lines, 22 out of 42 T_0_ plants were found to carry the mutant allele in *YGL1* homoeologous genes. Among these 22 gene-edited transgenic plants, TW181-36, TW181-3, and TW181-1 plants were homozygous or bi-allelic mutants at the edited sites of the 7A, 7B, and 7D genomes, respectively (**Supplementary Figure S2**, **Supplementary Table S2**), and these plants showed chlorotic leaf phenotype like the mutant *ygl1* (**Figure 5B**), indicating the three *YGL1* homoeologs have conserved functions. In addition, the seedlings of the plants (TW181-6, 34, 35, 43) with homozygous or bi-allelic mutants in all three genomes also showed chlorotic leaf phenotype and were shorter and weaker than other plants, suggesting that the function of the three genes had a dose effect (**Figure 5B**, **Supplementary Table S2**). The remaining plants that carried heterozygous mutations or the non-gene-edited plants showed green leaf phenotypes similar to that of the wild type. These results indicate that the targeted mutation co-segregated with the phenotypic variation of chlorosis leaf and demonstrate that *TraesCS7A02G480700* is the functional gene of *YGL1*.

### varBScore Can Identify Causal Mutations With Less Background Noise Than SNP-Index

**Figure 6:**
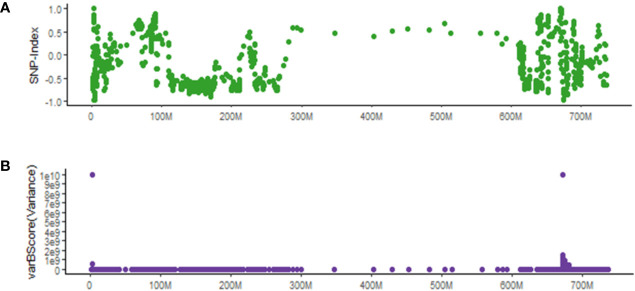
Comparison between varBScore and SNP-Index methods in identifying potential causal SNPs for the *ygl1* mutant. **(A)** △SNP-index bases on 20 SNP windows and five SNP steps. **(B)** The varBScore variance model based on 20 SNP windows and five SNP steps.

We compared varBScore with the popular SNP-Index method (Abe et al., 2012) in identifying potential causal SNPs for the *ygl1* mutant. As shown in **Figure 6A**, the △SNP-index has very dramatic changes in the multiple regions of 0–10 M, 90–200 M, and 600–700 M on chr7A. Due to the much higher noise level (several sites were close to 1 or −1) with SNP-Index, the SNP-Index method could not determine the candidate region. In contrast, the results with varBScore show only two distinct candidate segments (**Figure 6B**), and one of the two segments was the candidate gene target.

## Discussion

In the present study, we devised a BSA-Seq method based on a newly designed wheat exome capture panel and the varBScore algorithm and successfully applied it to clone the causal mutation in yellow-green leaf mutant *ygl1* in a much faster and efficient manner compared to the conventional map-based cloning strategy. Like other BSA-based cloning methods, our approach started with construction of a population derived from a cross between the isolated mutant line and a genetically divergent accession, followed by the use of an F_3_ population to bulk the green and yellow samples for exome capture sequencing with approximately 70× average depth. We then implemented a novel algorithm model, varBScore, and a series of bioinformatic analyses to fine-map *YGL1* within seven genes. Through functional annotation of these seven genes and validation of the gene function by the CRISPR/Cas9 method, we confirmed that the magnesium-chelatase subunit chlI encoded by *TraesCS7A02G480700* was the candidate gene carrying the causal mutation for yellow leaf phenotype of *ygl1*. Thus, BSE-Seq is a time- and cost-saving strategy for discovering the causal mutation responsible for the mutant phenotype.

Despite the considerable sequencing cost reduction due to the increasing data volume per sequencing run in different high-throughput sequencing platforms, it is still quite expensive to perform the genetic variation analyses in wheat by whole genome resequencing. In wheat, the over 90% sequence identity in coding regions from the A, B, and D homoeologous genomes as well as the high content of repetitive DNA and large retroelements has brought extra challenges for analyzing the large body of data (Uauy, 2017). Exons constitute approximately 0.95% of the wheat genome but code for the primary protein components that play essential roles in plant growth and development. Therefore, exome capture is an alternative method to effectively sequence coding regions with low cost in wheat. Our new wheat exome capture probe set with an accumulated probe length of 245 Mb can cover 277 Mb gene regions considering the capture of the flanking sequences including promoter regions, untranslated regions neighboring to the exons, and introns.

**Figure 7:**
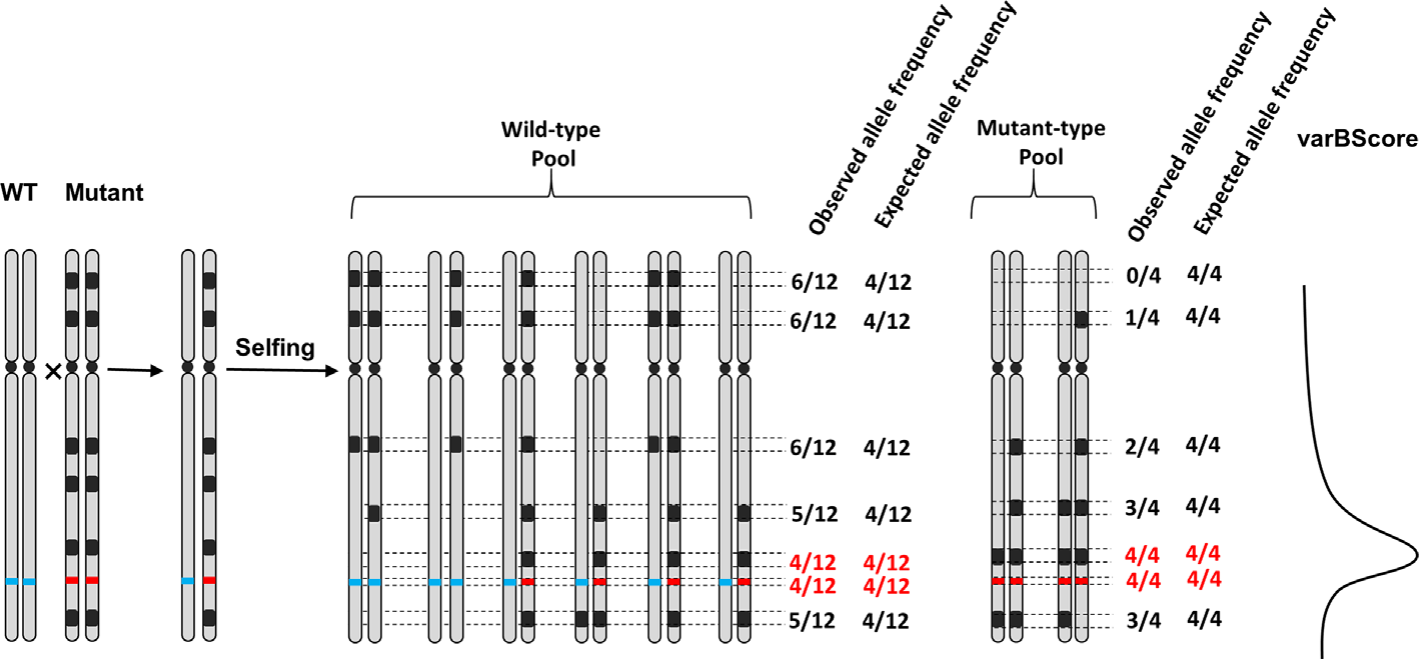
Schematic representation of the relationship among the observed allele frequency, expected allele frequency, and varBScore in the F_2_ population. Black regions represent the mutations in the mutant, the red and blue lines on the chromosome represent the genotypes of the mutant and wild-type at the candidate target sites, respectively. The observed and expected allele frequencies of mutant sites are calculated in the wild/mutant pool. The rightmost curve indicates the trend of varBScore. Regions where the observed allele frequency meets the expected allele frequency (marked with red color) in both the wild-type pool and mutant pools have a higher varBScore.

SNP-index and △SNP-index have been widely used for identifying potential causal SNPs. Compared to △SNP-index, which is directly obtained from the subtraction of observed allele frequency between bulked pools, the expected allele frequency is introduced as another parameter in varBScore algorithm, which calculates the standard deviation (SD) of the observed allele frequency and expected allele frequency, leading to a higher accuracy in evaluating the degree of variation. According to the linkage inheritance rule, molecular markers close to the gene of interest on a chromosome tend to be inherited together during meiosis, thus they may share a similar expected allele frequency with the gene of interest. Therefore, the minimum variation occurs only when the observed allele frequencies of the mutant and wild-type pool both meet their expected allele frequency simultaneously. By evaluating the logarithm of the variation, then the varBScore would be higher than those regions that do not satisfy the condition (**Figure 7**). As a result, the variances in the bulked pool that meet the expected allele frequency would show a peak in the varBScore plot. The advantage of the varBScore is that standard deviation was used to evaluate the variation, which would be more precise than the subtraction. This algorithm can reduce the background noise to some extent and provides an alternative method to identify causal mutations.

Gene-editing has emerged as a powerful tool for functional gene validation and mutant creation in both animals and plants (Chen et al., 2019). However, a problem that cannot be ignored is that there is a chance of off-target editing, which may introduce some unexpected phenotypes (Jin et al., 2019). Selecting specific target sites for editing is a better strategy to avoid or reduce the problem of off-target editing. To design specific target sites for gene editing, we aligned the sequence of the target locus with the wheat reference genome and found that the sequence did not significantly align with any other genomic regions in wheat, indicative of a relatively low possibility of off-target editing. As we expected, our observation of T_0_ plants carrying the homozygous mutations showed that the targeted mutation co-segregated with phenotypic variation of chlorosis leaf (**Figure 5B**, **Supplementary S2**). Therefore, the functional validation supports the hypothesis that *TraesCS7A02G480700* is the functional gene *YGL1*. As a hexaploid crop, many genes have three homoeologs present in each of the three wheat genomes of bread wheat. The three homoeologs could be functionally redundant or have additive effects. We showed that a mutation in any of them produced a yellow leaf phenotype like that of the *ygl1* mutant and simultaneous knock-out of all three homoeologs of *YGL1* not only conferred the yellow phenotype of the edited plant but also showed slower and weaker growth than the plant with only one of copy edited (**Figure 5B**). These results suggested that the three homoeologs share the same function but may also have additive effects.

Combining BSA and the exome sequence strategy could accelerate gene mapping, especially in polyploid species with large and complex genome such as wheat. First, although generating the segregant populations is still a rate-limiting step compared with technologies like MutRenSeq and AgRenSeq (Steuernagel et al., 2016; Arora et al., 2019), BSE-Seq could be easily used to identify the linked interval regardless of the multiple gene copies and high similarity among the homoeologs. An additional benefit is that whole-exome capture sequencing could obtain most of the variations existing in the coding regions of genes, which is helpful for the construction of linkage map across the whole genome. Specifically, capturing R genes leads to the reduction of data analysis and the cost of sequencing, while it carries the risk of missing of those non-typical NLRs resistance genes. In this sense, BSE-Seq has a wider range of adaptability. Secondly, compared with the 84-Mb and 110-Mb exome capture probe panels developed previously (Gardiner et al., 2016; Krasileva et al., 2017), the newly developed panel in our research or larger capture probe sets designed recently (Gardiner et al., 2019) could capture more sequence information, leading to a higher resolution in gene mapping and identification; even if the mutant gene was not captured, SNPs along the two flanks of the candidate gene would be sufficient to identify the linked interval. Thirdly, the varBSore algorithm raised here provides an alternative method to identify causal mutations and fits for various segregant populations including F_2_, F_2:3_, RILs (recombinant inbred lines), and DHs (doubled haploids), which may have a wider application in gene mapping and identification.

## Data Availability Statement

Customized exome capture design files and inhouse scripts will be made available by the authors upon request. Sequence data have been submitted to GenBank under Bioproject no. PRJNA579285.

## Author Contributions

XK, LZ, ZC, and XL conceived the study and designed the experiments. CX, CD, DL, ZX, QZ, and XZ, performed the experiments. ZC and LG performed the data analysis. JW provided the computing resources. YG, LZ, ZC, and XK wrote the manuscript. All authors contributed to the article and approved the submitted version.

## Funding

We thank Prof. Qixin Sun’s group for providing the CRISPR/Cas9 vector system. This work was supported by the National Transgenic Research Project (2016ZX08009001-001-004), the National Key Research and the Development Program of China (2016YFD0101001), the Agricultural Science and Technology Innovation Program of Chinese Academy of Agricultural Sciences (CAAS) and the Tianshan Innovation Team Plan (2020D14002).

## Conflict of Interest

Authors ZC and LG were employed by the company Chengdu Tcuni Technology.

The remaining authors declare that the research was conducted in the absence of any commercial or financial relationships that could be construed as a potential conflict of interest.
