# Supplementary material for: Combining a New Exome Capture Panel With an Effective varBScore Algorithm Accelerates BSA-Based Gene Cloning in Wheat
Source: Front Plant Sci. 2020 Aug 13;11:1249. doi: 10.3389/fpls.2020.01249 (PMC7438552; doi:10.3389/fpls.2020.01249)
Supplement: Supplementary file 1 [file Image_1.pdf]

Combining a new exome capture panel with an effective varBScore algorithm accelerates BSA-based gene cloning in wheat

Chunhao Dong[1,†], Lichao Zhang[1,†,*], Zhongxu Chen[2,3,†], Chuan Xia[1], Yongqiang Gu[5], Jirui Wang[3,4], Danping Li[1], Zhencheng Xie[1], Qiang Zhang[1], Xueying Zhang[1], Lixuan Gui[2,3], Xu Liu[1]*, Xiuying Kong[1]*

[1]Key Laboratory for Crop Gene Resources and Germplasm Enhancement, MOA, National Key Facility for Crop Gene Resources and Genetic Improvement, Institute of Crop Sciences, Chinese Academy of Agricultural Sciences, Beijing 100081, China

[2]Chengdu Tcuni Technology, Chengdu 610041, China

[3]State Key Laboratory of Crop Gene Exploration and Utilization in Southwest China, Sichuan Agricultural University, Chengdu 611130, China

[4]Triticeae Research Institute, Sichuan Agricultural University, Chengdu, 611130, China

[5]United States Department of Agriculture-Agricultural Research Service, Western Regional Research

*** Correspondence:**
Corresponding Author: Lichao Zhang (zhanglichao@caas.cn), Xu Liu (liuxu03@caas.cn) and Xiuying Kong(kongxiuying@caas.cn). Tel: 8610-82105828.

[†] These authors contributed equally to this work.

SUPPLEMENTARY MATERIAL

Supplementary Figure S1. Polygenetic tree for YGL1 and its homologues.
Supplementary Figure S2. Sequence of *YGL1* homoeologs at target site in gene-edited plants.
Supplementary Table S1. Genetic analysis of the mutant phenotypes of *ygl1*.
Supplementary Table S2. Statistics of *YGL1* and gene editing results.
Supplementary Table S3. PCR primers used for vector construction and editing identification in this study.

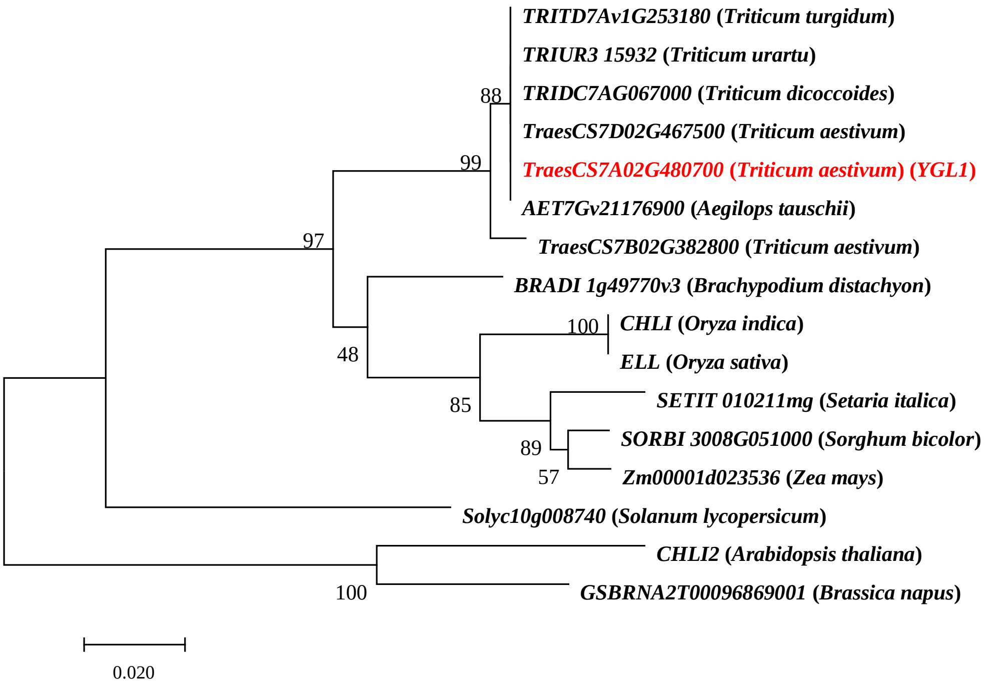

Supplementary Figure S1. Polygenetic tree for *YGL1* and its homologues. The polygenetic tree was constructed using the neighbor-joining method with MEG7.0 software. Numbers above the branches represent bootstrap support based on 1000 bootstrap replicates. Branch length represents substitutions per site. *YGL1* was indicated by red color.

TW181-1

```
Reference   TTGCCAAGGCCAACAGGGGGATACTGTATGTGGACGA
Genome D    TTGCCAAGGCCAACAGGGGGATA------TATGTGGACGA
Genome D    TTGCCAAGGCCAACAGGGGGATA---------TGTGGACGA
```

TW181-2

```
Reference   TCGCCAAGGCCAACAGGGGGATACTGTATGTGGACGA
Genome A    TCGCCAAGGCCAACAGGGGGATACTGTATGTGGACGA
Genome A    TCGCCAAGGCCAACA-------------------------TGTGGACGA

Reference   TTGCCAAGGCCAACAGGGGGATACTGTATGTGGACGA
Genome B    TTGCCAAGGCCAACAGGGGGATACT----ATGTGGACGA
Genome B    TTGCCAAGGCCAACAGGGGGATACT------------GGACGA

Reference   TTGCCAAGGCCAACAGGGGGATACTGTATGTGGACGA
Genome D    TTGCCAAGGCCAACAGGGGGATAC------ATGTGGACGA
Genome D    TTGCCAAGGCCAACAGGGGGATAC------ATGTGGACGA
```

TW181-3

```
Reference   TTGCCAAGGCCAACAGGGGGATACTGTATGTGGACGA
Genome B    TTGCCAAGGCCAACAGGGGGATA--------ATGTGGACGA
Genome B    TTGCCAAGGCCAACAGGGGGATAC----TATGTGGACGA
```

TW181-4

```
Reference   TCGCCAAGGCCAACAGGGGGATACTGT--ATGTGGACGA
Genome A    TCGCCAAGGCCAACAGGGGGATACTGTAATGTGGACGA
Genome A    TCGCCAAGGCCAACAGGGGGATACTGTTATGTGGACGA

Reference   TTGCCAAGGCCAACAGGGGGATACTGTATGTGGACGA
Genome B    TTGCCAAGGCCAACAGGGGGATAC--------TGTGGACGA
Genome B    TTGCCAAGGCCAACAGGGGGATA----------TGTGGACGA

Reference   TTGCCAAGGCCAACAGGGGGATACTGT--ATGTGGACGA
Genome D    TTGCCAAGGCCAACAGGGGGATACTGTTATGTGGACGA
Genome D    TTGCCAAGGCCAACAGG---------------------ATGTGGACGA
```

TW181-5

```
Reference   TCGCCAAGGCCAACAGGGGGATACTGT--ATGTGGACGA
Genome A    TCGCCAAGGCCAACAGGGGGATACT------ATGTGGACGA
Genome A    TCGCCAAGGCCAACAGGGGGATACTGTAATGTGGACGA

Reference   TTGCCAAGGCCAACAGGGGGATACTGTATGTGGACGA
Genome B    TTGCCAAGGCCAACAGGGGGA----------TATGTGGACGA
Genome B    TTGCCAAGGCCAACAGGGGGATACT---TATGTGGACGA
```

Reference TTGCCAAGGCCAACAGGGGGATACTGT--ATGTGGACGA
Genome D TTGCCAAGGCCAACAGGGGGATACTGT--ATGTGGACGA
Genome D TTGCCAAGGCCAACAGGGGGATACTGTAATGTGGACGA

TW181-6

Reference TCGCCAAGGCCAACAGGGGGATACTGTATGTGGACGA
Genome A TCGCCAAGGCCAACAGGGGGATAC----TATGTGGACGA
Genome A TCGCCAAGGCCAACAGGGGGATAC ATTATGTGGACGA

Reference TTGCCAAGGCCAACAGGGGGATACTGTATGTGGACGA
Genome B TTGCCAAGGCCAACAGGGGGATACTG--ATGTGGACGA
Genome B TTGCCAAGGCCAACAGGGG----------------ATGTGGACGA

Reference TTGCCAAGGCCAACAGGGGGATAC----TGTATGTGGACGA
Genome D TTGCCAAGGCCAACAGGGGGATA------------ATGTGGACGA
Genome D TTGCCAAGGCCAACAGGGGGATACAATGTATGTGGACGA

TW181-9

Reference TCGCCAAGGCCAACAGGGGGATACTGTATGTGGACGA
Genome A TCGCCAAGGCCAACAGGGGGATAC----TATGTGGACGA
Genome A TCGCCAAGGCCAACAGGGGGATA----------TGTGGACGA

Reference TTGCCAAGGCCAACAGGGGGATACTGTATGTGGACGA
Genome B TTGCCAAGGCCAACAGGGGGATAC----TATGTGGACGA
Genome B TTGCCAAGGCCAACAGGGGGATA--------ATGTGGACGA

Reference TTGCCAAGGCCAACAGGGGGATACTGTATGTGGACGA
Genome D TTGCCAAGGCCAACAGGGGGATACTGTATGTGGACGA
Genome D TTGCCAAGGCCAACAGGGGGATA----------TGTGGACGA

TW181-10

Reference TTGCCAAGGCCAACAGGGGGATACTGTATGTGGACGA
Genome D TTGCCAAGGCCAACAGGGGGATACTGTATGTGGACGA
Genome D TTGCCAAGGCCAACAGGGGGATA-------TATGTGGACGA

TW181-11

Reference TTGCCAAGGCCAACAGGGGGATACTGTA--TGTGGACGA
Genome B TTGCCAAGGCCAACAGGGGGATACTGTA--TGTGGACGA
Genome B TTGCCAAGGCCAACAGGGGGATACTGTAATGTGGACGA

Reference TTGCCAAGGCCAACAGGGGGATACTGTATGTGGACGA
Genome D TTGCCAAGGCCAACAGGGGGATACTGTATGTGGACGA
Genome D TTGCCAAGGCCAACAGG--------------------TGTGGACGA

TW181-20

Reference	TTGCCAAGGCCAACAGGGGGATACTGTATGTGGACGA
Genome D	TTGCCAAGGCCAACAGGGGGATACTGTATGTGGACGA
Genome D	TTGCCAAGGCCAACAGGGGGATA----------TGTGGACGA

TW181-22

Reference	TCGCCAAGGCCAACAGGGGGATACTGTATGTGGACGA
Genome A	TCGCCAAGGCCAACAGGGGGATACTGTATGTGGACGA
Genome A	TCGCCAAGGCCAACAGGGGGATACT----ATGTGGACGA

Reference	TTGCCAAGGCCAACAGGGGGATACTGTATGTGGACGA
Genome B	TTGCCAAGGCCAACAGGGGGA-----------ATGTGGACGA
Genome B	TTGCCAAGGCCAACAGGGGGA-----------ATGTGGACGA

TW181-23

Reference	TCGCCAAGGCCAACAGGGGGATACTG--TATGTGGACGA
Genome A	TCGCCAAGGCCAACAGGGGGATACTGTTATGTGGACGA
Genome A	TCGCCAAGGCCAACAGGGGGATA-------------GTGGACGA

Reference	TTGCCAAGGCCAACAGGGGGATACTGTATGTGGACGA
Genome B	TTGCCAAGGCCAACAGGGGGATA------TATGTGGACGA
Genome B	TTGCCAAGGCCAACAGGGGGA-----------ATGTGGACGA

Reference	TTGCCAAGGCCAACAGGGGGATACTGTATGTGGACGA
Genome D	TTGCCAAGGCCAACAGGGGGATACTGTATGTGGACGA
Genome D	TTGCCAAGGCCAACAGGGGGATAC---------------------GA

TW181-24

Reference	TCGCCAAGGCCAACAGGGGGATACTGTATGTGGACGA
Genome A	TCGCCAAGGCCAACAGGGGGATA----------TGTGGACGA
Genome A	TCGCCAAGGCCAACAGGGGGATA----------TGTGGACGA

Reference	TTGCCAAGGCCAACAGGGGGATACTGTATGTGGACGA
Genome B	TTGCCAAGGCCAACAGGGGGATAC----TATGTGGACGA
Genome B	TTGCCAAGGCCAACAGGGGGATA--------ATGTGGACGA

TW181-26

Reference	TCGCCAAGGCCAACAGGGGGATACTGTATGTGGACGA
Genome A	TCGCCAAGGCCAACAGGGGGATACTGTATGTGGACGA
Genome A	TCGCCAAGGCCAACAGGGGGATA------TATGTGGACGA

Reference	TTGCCAAGGCCAACAGGGGGATACTGTATGTGGACGA
Genome B	TTGCCAAGGCCAACAGGGGGATACTGTATGTGGACGA
Genome B	TTGCCAAGGCCAACAGGGG--A-----------ATGTGGACGA

Reference	TTGCCAAGGCCAACAGGGGGATACTGTATGTGGACGA
Genome D	TTGCCAAGGCCAACAGGGGGATACTGTATGTGGACGA

Genome D   TTGCCAAGGCCAACAGGGGGATA----------TGTGGACGA

TW181-30

Reference   TTGCCAAGGCCAACAGGGGGATACTGTATGTGGACGA
Genome B   TTGCCAAGGCCAACAGGGGGATA------TATGTGGACGA
Genome B   TTGCCAAGGCCAACAGGGGGAT-----------TGTGGACGA

Reference   TTGCCAAGGCCAACAGGGGGATACTGTATGTGGACGA
Genome D   TTGCCAAGGCCAACAGGGGGATACTGTATGTGGACGA
Genome D   TTGCCAAGGCCAACAGGGGGATAC-------------------CGA

TW181-31

Reference   TTGCCAAGGCCAACAGGGGGATACTGTATGTGGACGA
Genome B   TTGCCAAGGCCAACAGGGGGATACTGTATGTGGACGA
Genome B   TTGCCAAGGCCAACAGGGGGATACTGTATGTGGA----A

Reference   TTGCCAAGGCCAACAGGGGGATACTGTATGTGGACGA
Genome D   TTGCCAAGGCCAACAGGGGGATACTGTATGTGGACGA
Genome D   TTGCCAAGGCCAACAGGG--------------GTATGTGGACGA

TW181-34

Reference   TCGCCAAGGCCAACAGGGGGATACTGTATGTGGACGA
Genome A   TCGCCAAGGCCAACAGGGGGATACTGTTTGTGGACGA
Genome A   TCGCCAAGGCCAACAGGGGGAT----------ATGTGGACGA

Reference   TTGCCAAGGCCAACAGGGGGATACTGTATGTGGACGA
Genome B   TTGCCAAGGCCAACAGGGGGAT----------ATGTGGACGA
Genome B   TTGCCAAGGCCAACAGGGGGAT--------------GTGGACGA

Reference   TTGCCAAGGCCAACAGGGGGATACTG--TATGTGGACGA
Genome D   TTGCCAAGGCCAACAGGGGGATACTGTTATGTGGACGA
Genome D   TTGCCAAGGCCAACAGGGGGAT----------TATGTGGACGA

TW181-35

Reference   TCGCCAAGGCCAACAGGGGGATACTGT--ATGTGGACGA
Genome A   TCGCCAAGGCCAACAGGGGGATACTGTTATGTGGACGA
Genome A   TCGCCAAGGCCAACAGGGGGATACTGTGTGTGGACGA

Reference   TTGCCAAGGCCAACAGGGGGATACTGTATGTGGACGA
Genome B   TTGCCAAGGCCAACAGGGGGAT--------TATGTGGACGA
Genome B   TTGCCAAGGCCAACAGGGGGATACTGTGTGGGGACGA

Reference   TTGCCAAGGCCAACAGGGGGATACTGTATGTGGACGA
Genome D   TTGCCAAGGCCAACAGGGGGATACT----ATGTGGACGA
Genome D   TTGCCAAGGCCAACAGGGGGATACT--TATGTGGACGA

TW181-36

Reference	TCGCCAAGGCCAACAGGGGGATACTGTATGTGGACGA
Genome A	TCGCCAAGGCCAACAGGGGGATA--------ATGTGGACGA
Genome A	TCGCCAAGGCCAACAGGGGGATA--------ATGTGGACGA

TW181-41

Reference	TCGCCAAGGCCAACAGGGGGATACTGTATGTGGACGA
Genome A	TCGCCAAGGCCAACAGGGGGATACTGTATGTGGACGA
Genome A	TCGCCAAGGCCAACAGGGGGATACTG-------------ACGA

Reference	TTGCCAAGGCCAACAGGGGGATACTGTATGTGGACGA
Genome B	TTGCCAAGGCCAACAGGGGGATAC-------------GGACGA
Genome B	TTGCCAAGGCCAACAGGG--------------------GTGGACGA

Reference	TTGCCAAGGCCAACAGGGGGATACTGT--ATGTGGACGA
Genome D	TTGCCAAGGCCAACAGGGGGATACTGTAATGTGGACGA
Genome D	TTGCCAAGGCCAACAGGG------------------ATGTGGACGA

TW181-42

Reference	TCGCCAAGGCCAACAGGGGGATACTGT--ATGTGGACGA
Genome A	TCGCCAAGGCCAACAGGGGGATACTGTTATGTGGACGA
Genome A	TCGCCAAGGCCAACAGGGGGATACTGTTATGTGGACGA

Reference	TTGCCAAGGCCAACAGGGGGATACTGTATGTGGACGA
Genome B	TTGCCAAGGCCAACAGGGGGATACTGTATGTGGACGA
Genome B	TTGCCAAGGCCAACAGGGGGATACT--TATGTGGACGA

Reference	TTGCCAAGGCCAACAGGGGGATACTGTATGTGGACGA
Genome D	TTGCCAAGGCCAACAGGGGGA------------TGTGGACGA
Genome D	TTGCCAAGGCCAACAG---------------------------GGACGA

TW181-43

Reference	TCGCCAAGGCCAACAGGGGGATACTGTATGTGGACGA
Genome A	TCGCCAAGGCCAACAGGGGGATACT--TATGTGGACGA
Genome A	TCGCCAAGGCCAACAGGGGGATACT--TATGTGGACGA

Reference	TTGCCAAGGCCAACAGGGGGATACTGTATGTGGACGA
Genome B	TTGCCAAGGCCAACAGGGGGATA----------TGTGGACGA
Genome B	TTGCCAAGGCCAACAGGGGGATA----------TGTGGACGA

Reference	TTGCCAAGGCCAACAGGGGGATACTGT--ATGTGGACGA
Genome D	TTGCCAAGGCCAACAGGGGGATACTGTTATGTGGACGA
Genome D	TTGCCAAGGCCAACAGGGGGATACTGTTATGTGGACGA

Supplementary Figure S2. Sequence of *YGL1* homoeologs at target site in gene-edited plants. Nucleotide sequences in red show the sgRNA region; sequence underlined indicates the PAM

sequence, black dashes in sequence represent the deletions, and letters in blue represent insertion or substitution.

Supplementary Table S1. Genetic analysis of the mutant phenotypes of *ygl1*

Cross combination	F_1		F_2		χ^2 (3:1)	P-value
	Wild type	*ygl1*	Wild type	*ygl1*		
YZ4110/*ygl1*	56	0	930	247	3.23	0.072
Bainong3217/*ygl1*	15	0	329	102	0.41	0.552

Supplementary Table S2. Statistics of *YGL1* and gene editing results

Plants	Leaf color	Genotype of *TraesCS7A02G480700*	Genotype of *TraesCS7B02G382800*	Genotype of *TraesCS7D02G467500*
TW181-1	Yellow	WT / WT	WT / WT	5bp deletion / 3bp deletion
TW181-2	Yellow	WT / 13bp deletion	6bp deletion/ 2bp deletion	3bp deletion / 3bp deletion
TW181-3	Yellow	WT / WT	4bp deletion / 2bp deletion	WT / WT
TW181-4	Yellow	1bp insertion / 1bp insertion	5bp deletion / 4bp deletion	10bp deletion / 1bp insertion
TW181-5	Yellow	2bp deletion/ 1bp insertion	5bp deletion/ 1bp deletion	WT/1bp insertion
TW181-6	Yellow	2bp deletion / AT to TG substitution	1bp deletion / 8bp deletion	4bp deletion / 2bp insertion
TW181-7	Green	WT / WT	WT / WT	WT / WT
TW181-8	Green	WT / WT	WT / WT	WT / WT
TW181-9	Yellow	2bp deletion / 5bp deletion	4bp deletion / 2bp deletion	WT / 5bp deletion
TW181-10	Green	WT / WT	WT / WT	WT / 3bp deletion
TW181-11	Green	WT / WT	WT / 1bp insertion	WT / 11bp deletion

Plants	Leaf color	Genotype of *TraesCS7A02G480700*	Genotype of *TraesCS7B02G382800*	Genotype of *TraesCS7D02G467500*
TW181-12	Green	WT / WT	WT / WT	WT / WT
TW181-13	Green	WT / WT	WT / WT	WT / WT
TW181-14	Green	WT / WT	WT / WT	WT / WT
TW181-15	Green	WT / WT	WT / WT	WT / WT
TW181-16	Green	WT / WT	WT / WT	WT / WT
TW181-17	Green	WT / WT	WT / WT	WT / WT
TW181-18	Green	WT / WT	WT / WT	WT / WT
TW181-19	Green	WT / WT	WT / WT	WT / WT
TW181-20	Green	WT / WT	WT / WT	WT / 5bp deletion
TW181-21	Green	WT / WT	WT / WT	WT / WT
TW181-22	Yellow	WT / 2bp deletion	6bp deletion/ 6bp deletion	WT / WT
TW181-23	Yellow	6bp deletion / 1bp insertion	6bp deletion / 3bp deletion	WT / 11bp deletion

**Plants**	**Leaf color**	**Genotype of *TraesCS7A02G480700***	**Genotype of *TraesCS7B02G382800***	**Genotype of *TraesCS7D02G467500***
TW181-24	Yellow	5bp deletion / 5bp deletion	4bp deletion/ 2bp deletion	WT
TW181-25	Green	WT / WT	WT / WT	WT / WT
TW181-26	Green	WT / 3bp deletion	WT / 7bp deletion	WT / 5bp deletion
TW181-27	Green	WT / WT	WT / WT	WT / WT
TW181-28	Green	WT / WT	WT / WT	WT / WT
TW181-29	Green	WT / WT	WT / WT	WT / WT
TW181-30	Yellow	WT / WT	3 bp deletion/6 bp deletion	WT / 10bp deletion
TW181-31	Green	WT / WT	WT/2bp deletion	WT / 7bp deletion
TW181-32	Green	WT / WT	WT / WT	WT / WT
TW181-33	Green	WT / WT	WT / WT	WT / WT
TW181-34	Yellow	5bp insertion/ T to A substitution	5bp deletion / 7bp deletion	4bp deletion / 1bp insertion
TW181-35	Yellow	1bp insertion/ 1bp insertion	4bp deletion / G to A, G to T substitution	2bp deletion / 1 bp deletion

**Plants**	**Leaf color**	**Genotype of *TraesCS7A02G480700***	**Genotype of *TraesCS7B02G382800***	**Genotype of *TraesCS7D02G467500***
TW181-36	Yellow	4bp deletion / 4bp deletion	WT / WT	WT / WT
TW181-37	Green	WT / WT	WT / WT	WT / WT
TW181-38	Green	WT / WT	WT / WT	WT / WT
TW181-39	Green	WT / WT	WT / WT	WT / WT
TW181-41	Yellow	WT/7bp deletion	11bp deletion / 7bp deletion	9bp deletion / 1bp insertion
TW181-42	Yellow	1bp insertion/ 1bp insertion	WT / 1bp deletion	7bp deletion / 15bp deletion
TW181-43	Yellow	1bp deletion/1bp deletion	5bp deletion / 5bp deletion	1bp insertion / 1bp insertion

Supplementary Table S3. PCR primers used for vector construction and editing identification in this study.

Primer name	Primer sequence (from 5' to 3')	Application	Note
ygl-MT1T2-F	aataatggtctcaagcgaCAACAGGGGGATACTGTATG gttttagagctagaaatagc	CRISPR/Cas9 construction for *YGL1* and its homeologous	The capital letters are oligoes for gRNA guide sequences, the lower letters with red color are the overhangs for sub-cloning
ygl-MT1T2-R	attattggtctctaaacCATACAGTATCCCCCTGTTG tcgcttcttggtgcc		
ygl-413Test-7A F1	ACATCGTCCGGCCTATCCAAGTC	PCR amplification for *TraesCS7A02G480700* target site	
ygl-413Test-7A R1	GTCACGATGTCCCTTCCCTTTAG		
ygl-413Test-7B F1	GCACCCTTAAAAAAATCGGAGAAG	PCR amplification for *TraesCS7B02G382800* target site	
ygl-413Test-7B R1	TGGCTGCACAAGTGCATTTTCT		
ygl-413Test-7D F1	TTTTGTCATGTTTTTTTAGGCATAC	PCR amplification for *TraesCS7D02G467500* target site	
ygl-413Test-7D R1	TGGCTCCACAAGTGCATTTTCTT		
ygl-7A-Seq F1	ACCACCACCAAGATCACCATG	Sequencing primer for *TraesCS7A02G480700* target site	
ygl-7B-Seq F1	CCTCAAGGGCGAGGACCTTA	Sequencing primer for *TraesCS7B02G382800* target site	
ygl-7D-Seq F1	CCTCAAGGGCGAGGACCTTC	Sequencing primer for *TraesCS7D02G467500* target site	
